# Controlling Lyme Disease: New Paradigms for Targeting the Tick-Pathogen-Reservoir Axis on the Horizon

**DOI:** 10.3389/fcimb.2020.607170

**Published:** 2020-12-03

**Authors:** Quentin Bernard, James P. Phelan, Linden T. Hu

**Affiliations:** Department of Molecular Biology and Microbiology, Tufts University School of Medicine, Boston, MA, United States

**Keywords:** tick-borne diseases, prevention, *Borrelia burgdorferi*, *Ixodes scapularis*, reservoir host

## Introduction

Lyme disease is caused by a bacterium, *Borrelia burgdorferi* (*B. burgdorferi*), and is transmitted by an acarian vector, *Ixodes* ticks (Radolf et al., 2012). As the most common vector-borne disease in the northern hemisphere, it is spread in at least 80 countries. In the United States, it affects an estimated 300,000 people every year and costs the US economy up to 3 billion dollars per year (Adrion et al., 2015). Developing preventive strategies against this disease is critical in reducing its negative impact on people’s health and countries’ economies. While there are ongoing efforts toward prevention in humans, these will not affect the reservoirs of disease as humans are an accidental host of *B. burgdorferi* and not important for maintenance of the bacteria in the wild. As such, human targeted interventions such as vaccines, insect repellents, and prophylactic treatments require continual investments as they do not reduce the risk of infection beyond the person using the intervention. For long-term control of tick-borne infections, it is necessary to go to the source.

Rodents, especially *Peromyscus leucopus* in north America, are the main reservoir host of *B. burgdorferi* (Wilson et al., 1985). In Europe, birds are also largely involved in the bacteria life cycle notably of *B. garinii* (Humair et al., 1998). Interventions targeting the vector or these reservoir hosts have the potential to alter the trajectory of the disease permanently, both in incidence and geographic distribution. Here, we will review some of the more promising approaches to interfere with the life cycle of *B. burgdorferi*.

## Vector Focused Approaches

### Acaricides

The tick is the bridge for vector-borne pathogens, including *B. burgdorferi*, that allows them to transit from reservoir hosts to human. An advantage of targeting the vector rather than a specific pathogen is that it has the potential to reduce all diseases transmitted by that vector. Reduction of local tick populations through the application of acaricides has been recommended by experts and is available through many pest control companies (Curran et al., 1993). However, a study by Hinckley et al. has suggested that, although environmental spraying of acaricides is effective at reducing ticks around domiciles with a >60% reduction in questing ticks, it had no effect on Lyme disease transmission to the homeowners when used on their property only (Hinckley et al., 2016). One possible explanation is that the homeowners using acaricides are acquiring Lyme disease from ticks outside their property. In support of this possibility, the risk of getting infected at the neighborhood scale has been shown to be 57% greater than the risk at the yard scale (Fischhoff et al., 2019).

More targeted applications of acaricides have also been tested and have the advantage of decreasing environmental effects by limiting the distribution of the agents. It was shown that the treatment of white-tailed deer with topical acaricides such as fipronil or permethrin using a “four-poster device” can help control the *l. scapularis* population. Although deer are not competent reservoir hosts for *B. burgdorferi*, they are important in the *Ixodes* tick life cycle. Use of the four-poster device resulted in 46%–70% reduction in nymphal ticks (Stafford et al., 2009). Limited data suggest it may also have an effect on human Lyme disease (Garnett et al., 2011). Notably, a targeted acaricidal approach had a greater effect on human Lyme disease cases than deer reduction through hunting. Deer reduction through contraception—either through hormones or with a vaccine—is also being explored (Rutberg et al., 2013). However, this practice can have short and long-term negative impact on animal welfare including physiological changes, altered behavior and extended breeding seasons that would require additional study before widespread deployment (Hampton et al., 2015).

Targeting of ticks on mice using acaricides has also been utilized and products are commercially available. Three strategies have been employed: bait boxes which coat the mice as they enter a feeding station, nesting material impregnated with an acaricide that transfers the agent to mouse fur in the nest or oral feeding with acaricide-containing baits. Each has been effective at reducing ticks on the mice; however, data for prevention of human disease are mixed (White and Gaff, 2018).

Development of resistance to acaricides is one of the limitations of any acaricidal strategy. A recent study has shown that 50% of *Rhipicephalus microplus* ticks, a tick infesting cattle in United States, were resistant to permethrin in 2017 as opposed to 3% in 2008 in the same tested area (Thomas et al., 2020). Moreover, acaricides can be highly toxic to various animals in the environment. Benefits associated to acaricides must always be weighed against impacts on the environment including non-target insects and toxicity to humans (van Wieren et al., 2016).

### Biopesticides

Variants of the acaricide strategy include the use of natural agents as a less toxic approach to killing ticks. Other natural acaricide and repellents, including different essential oils, garlic, and nootkatone, have been developed as more environmentally friendly agents but suffer from either short time of action or expense in production (Jordan et al., 2012; Nchu et al., 2016; Machtinger and Li, 2017; Faraone et al., 2019). Alternative approaches to directly killing ticks include molecules that can interfere with mating, using sex pheromones for example, or that gather pests into traps. Control of the dog tick *Rhipicephalus sanguineus* using a gold nanoparticle assembly of a pheromone complex as a bait or vapor patches dispersing pheromones have been successfully tested (Anish et al., 2017; Gowrishankar et al., 2019). No similar products for *Ixodes* ticks have been developed to date.

### Biocontrol

Microbial controls for ticks that have been studied include natural and engineered fungi, bacteria and viruses that kill ticks. The fungi *Metarhizium brunneum* is an entomopathogenic fungus that has been shown to kill a variety of insects and arachnids, including *Ixodes* (Bharadwaj and Stafford, 2010). It was originally isolated from moths (Bischoff et al., 2009). Studies suggest that it is as effective as chemical acaricides but with less of an impact on non-target species (Bharadwaj and Stafford, 2010; Fischhoff et al., 2017). This fungus is now available commercially as Met52 and has been tested as part of integrated tick management strategies with some moderate effect (Williams et al., 2018). Entomopathogenic nematodes are also being considered against *Rhipicephalus microplus* as they can reduce oviposition, egg production index and larval hatching (de Mendonça et al., 2019). *Ixodiphagus hookeri*, a parasitoid wasp specialized in parasitizing larval and nymphal stages of *Ixodes* ticks could also be used as a biocontrol tool to against tick-transmitted pathogen (Krawczyk et al., 2020). Less developed agents include the bacterium, *Bacillus thuringiensis* (Bt), widely used in agriculture to manage different insect species. It has been shown to have toxicity to *Ixodes* and *Dermacentor* ticks (Szczepańska et al., 2018). Viruses against *Ixodes* ticks have not been developed to date but a baculovirus genetically engineered to express a chitinase can kill *Haemaphysalis longicornis* ticks (Assenga et al., 2006). Genetically engineered baculoviruses have been widely used in agriculture for control of insects and are felt to be environmentally safe (Szewczyk et al., 2006). However, the most common baculoviruses do not infect ticks.

## Anti-Tick Vaccines

*Ixodes* ticks often carry multiple pathogens. A vaccine that would target the vector could prevent multiple diseases at the same time. Research using tick antigens to prevent successful tick feeding have been shown to be effective in the laboratory. A commercial vaccine against Bm86 protein from *Boophilus* ticks has been used to successfully protect cows against tick feeding (Fragoso et al., 1998). Several *Ixodes* proteins, including but not limited to subolesin, salivary proteins, tick salivary lectin pathway inhibitor, tick histamine release factor have shown promise as potential vaccines (Schuijt et al., 2011; Bensaci et al., 2012; Narasimhan et al., 2020). However, their effectiveness in preventing transmission of disease has been weak to moderate. The high evolutionary pressure exerted on some of these proteins during co-evolution of the tick and its natural hosts might make development of these targets more difficult than for non-natural hosts such as humans. The combination of several moderately effective antigens with different functions could improve efficacy of anti-tick vaccination (Rego et al., 2019).

## Reservoir Targeted Strategies

**Antibiotics**. The use of reservoir targeted antibiotics has been one of the most highly effective strategies for reducing carriage of tick-borne pathogens. Deployment of doxycycline hyclate containing baits targeting mice reduced the percentage of *B. burgdorferi* infected small mammals in treated areas by 89.6 percent and the infection rate in nymphal ticks by 94.3% following 2 years of treatment (Dolan et al., 2011). In addition, carriage of another pathogen, *Anaplasma phagocytophilum* was reduced 74% and 92% in mice and nymphal ticks respectively. While these results are very promising, the employment of a doxycycline-based strategy is complicated by concerns over development of antibiotic resistance. While *B. burgdorferi* have not been shown to evolve resistance to doxycycline, doxycycline is the only drug available to treat *Anaplasma* infections and another tick-borne infection, Rocky Mountain spotted fever. In addition, it is one of just a few oral agents active against methicillin resistant *Staphylococcus aureus* and there are concerns that widespread distribution could lead to development of resistance in non-target bacteria. Substitution of more narrow spectrum antibiotics that are not critical for treatment of human diseases may alleviate these concerns.

### Reservoir-Targeted Vaccination

Vaccines are an important weapon in the prevention of many diseases in humans. While there was previously a human vaccine for Lyme, it has been off the market since 2002. There are now efforts to bring to market newer versions of this vaccine, based on a recombinant version of the *B. burgdorferi* outer surface protein A (OspA) (Comstedt et al., 2017). OspA is fairly stable in U.S. strains but variations in OspA in European strains have led to the development of multivalent OspA vaccines for use in Europe (Comstedt et al., 2017; Nayak et al., 2020). Proteins other than OspA have been examined as potential vaccine candidates (Yang et al., 2010; Floden et al., 2011; Kung et al., 2016), but to date, only a multivalent outer surface protein C construct, approved for vaccination of dogs, is under consideration for a human vaccine (Izac et al., 2020). OspC is a highly variable protein with protection only against isogenic strains in studies. The dog OspA-OspC combination vaccine uses a chimeritope that induces antibodies reacting against 25 different recombinant OspC variants (Marconi et al., 2020). However, regardless of how effective a human vaccine is, it will not decrease the spread of the disease and will require a commitment to continue to vaccinate at risk individuals.

Repurposing the human OspA vaccine for use in wild reservoirs has been shown to decrease infection among nymphal ticks when given subcutaneously to *Peromyscus* mice in the prior year (Tsao et al., 2004). Subsequent studies have attempted to use oral routes of delivery for the OspA vaccine with either recombinant protein or a viral vector. However, the recombinant protein has proven to have low immunogenicity and the use of live viral vectors has raised environmental concerns. Deployment of recombinant OspA vaccines have shown possible reductions in tick infections with *B. burgdorferi* although the effect on seroconversion is modest (Richer et al., 2014; Stafford et al., 2020).

## The Future?

### Genetic Engineering for Reservoir and Vector Incompetence

The advent of CRISPR/Cas technologies have made targeted engineering of mice feasible. By incorporating the CRISPR/Cas machinery into an engineered cassette called a “gene-drive” and inserting it into the chromosome, animals can pass along the engineered trait in a dominant, non-Mendelian pattern as the inserted gene will create a copy of itself onto the other chromosome. Investigators have proposed using this tool to create *B. burgdorferi* resistant mice carrying a gene to express an anti-OspA antibody (Buchthal et al., 2019). Proof of principle studies are being carried out in mice without the use of the gene drive, but ultimately, use of gene drive technology could prove to be a low-cost mechanism for changing reservoir competence of an entire population of mice. However, the path to using this technology will be difficult due to concerns about the ability to control unforeseen events caused by a self-replicating, engineered mutation.

A similar genetic engineering strategy could be undertaken with a focus on ticks. Engineering ticks to be of a single sex to reduce tick populations or elimination of specific proteins required for vector competence (ability to acquire and transmit a pathogen) could be an effective strategy for controlling tick transmitted diseases. Similar strategies have been proposed for mosquitos in the control of dengue virus (Buchman et al., 2020). However, *Ixodes* tick biology with its 2-year cycle, may make tick gene drive approaches very challenging.

### Tick Immunity and the Microbiome

The role of the tick and vertebrate host microbiome on transmission of organisms is just beginning to be understood (Shaw and Catteruccia, 2019). Studies in mosquitoes have associated the human skin microbiome with susceptibility to bites by the insects (Verhulst et al., 2011). Specific metabolites released by the microbiome are able to attract mosquitoes (Busula et al., 2017). Repellents based on modulating the skin microbiome for insect repellency are already in development for *Triatoma infestans* and *Rhodnius prolixus* (Ramírez et al., 2020), two vectors of Chagas disease. Whether similar effects to repel *Ixodes* ticks could be engendered by changes in the mouse microbiome is unknown.

The tick immune system plays an important role in shaping the tick microbiome. Recently, a tick protein called PIXR, has been shown to alter the gut microbiome, metabolome and immune responses (Narasimhan et al., 2017). Interestingly, these alterations influence the spirochete persistence in the tick. Similarly, *Anaplasma phagocytophilum*, another pathogen transmitted by ticks, has been shown to induce a tick protein, *Ixodes scapularis* antifreeze glycoprotein (iafgp), in order to efficiently colonize the tick through a mechanism that perturbs the tick gut microbiome (Abraham et al., 2017). Altering the tick microbiome could potentially be a way to disrupt the tick gut peritrophic membrane and to stop the *B. burgdorferi* transmission (Narasimhan et al., 2014).

The co-evolution between *B. burgdorferi* and *Ixodes* ticks lead to a system in which the bacteria is able to some extent to hide from the tick immune system. While the tick is still able to respond to *B. burgdorferi* through different immune pathways (Smith et al., 2016; Kitsou and Pal, 2018), the bacteria may have developed ways to avoid inducing specific immune mechanisms. For example, hemocytes from *Dermacentor variabilis* ticks, an immunocompetent tick for *B. burgdorferi*, have been shown to be highly efficient at clearing the spirochete but not the ones from its natural vector *Ixodes scapularis* (Johns et al., 2001).

### Targeting Nutritional Vulnerabilities of *B. burgdorferi*

*B. burgdorferi* has a very spare genome (Fraser et al., 1997) and is highly dependent upon its environment for providing critical nutrients. For example, *B. burgdorferi* is not able to synthesize fatty acids and cholesterol, and rely completely on host lipids notably present in the blood meal. Moreover, it manipulates the production level of specific amino-acids in the tick, suggesting a critical need for the bacteria to highjack the tick metabolism pathways (Hoxmeier et al., 2017; Cabezas-Cruz et al., 2019). This creates unique vulnerabilities of the organism to the lack of specific nutrients and/or may allow targeting of pathways that are redundant in other bacteria, but limited in *B. burgdorferi*. Chakraborti et al. have shown that targeting the purine salvage pathway of *B. burgdorferi* with inhibitory nucleoside analogs resulted in killing of the organism (Chakraborti et al., 2020).

## Summary

**Figure 1:**
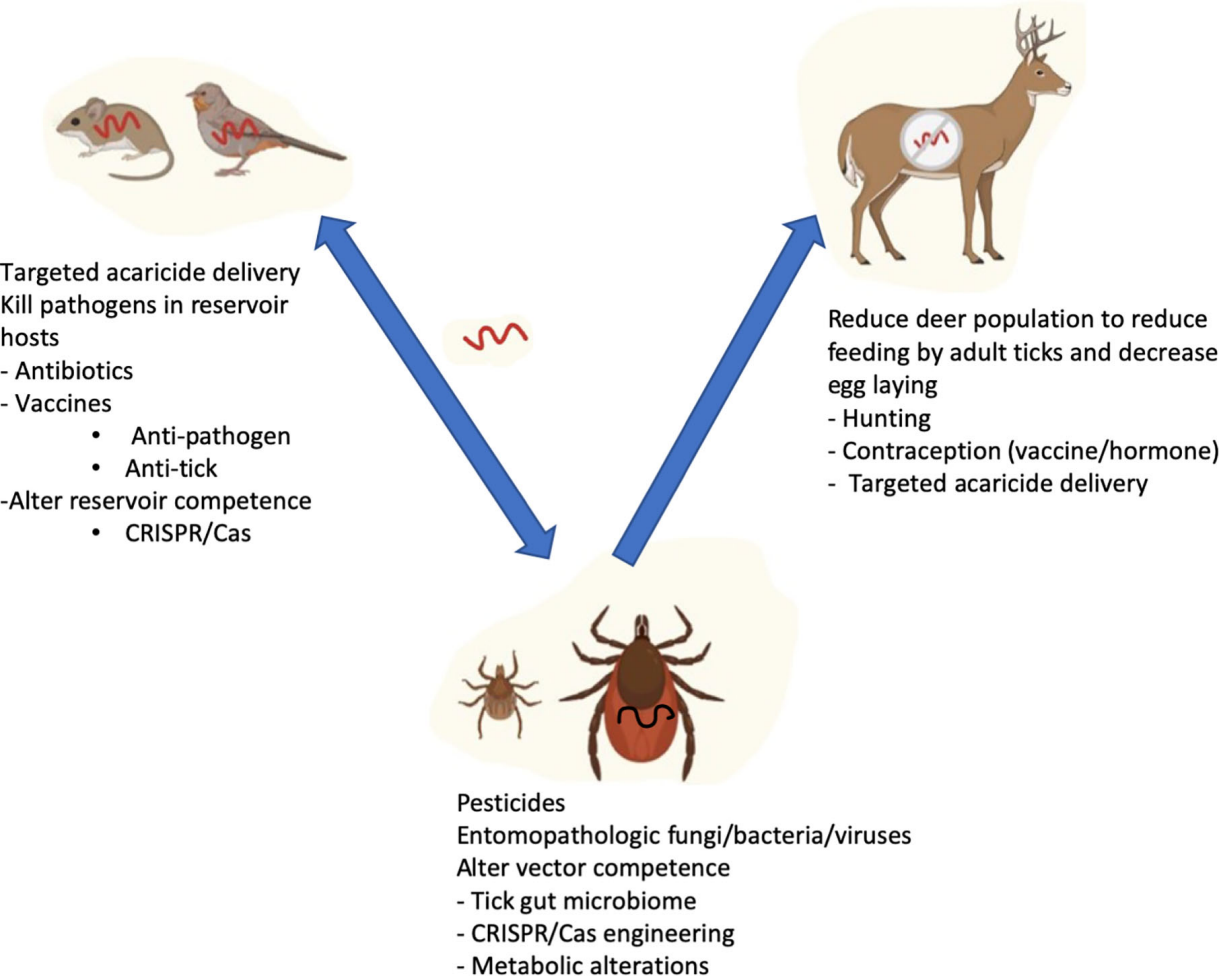
Strategies targeting reservoir host, ticks, and human host to prevent Lyme disease transmission. *B. burgdorferi* is perpetuated in a life cycle involving *Ixodes* ticks and reservoir hosts (Rodents and birds). Adult ticks preferably feed on bigger mammals such as deer which do not get infected with *B. burgdorferi*, but are very important for maintaining tick numbers. Humans are incidental hosts not important for perpetuation of the bacteria. Reduction of disease in the vector and reservoir hosts has the potential for reducing human infection.

The complexity behind triad of components (the pathogen, reservoir hosts, and the vector) involved in the transmission of vector-borne diseases to humans offers the opportunity to develop prevention strategies at multiple levels (**Figure 1**). The different strategies are not mutually exclusive, and it is likely that an effective strategy for reduction of tick-borne disease will need to employ multiple approaches. Continued advances in the understanding of the intersections of between the pathogen and reservoir host genomes, the impact of the microbiome and the development of tools such as gene editing provide some of the best hope that we will be able to control tick-borne infections in the future.

## Author Contributions

QB, JP, and LH wrote the manuscript. All authors contributed to the article and approved the submitted version.

## Funding

This work was supported by National Institutes of Health (NIH) (R01 AI152210, R21AI146841) and the Deborah and Charles Blackman-GLA Fellowship.

## Conflict of Interest

The authors declare that the research was conducted in the absence of any commercial or financial relationships that could be construed as a potential conflict of interest.
